# Negative Correlation between Serum NLRP3 and the Ratio of Treg/Th17 in Patients with Obstructive Coronary Artery Disease

**DOI:** 10.31083/j.rcm2312403

**Published:** 2022-12-12

**Authors:** ZhiXing Fan, YiFan Huang, JinChun Wu, ChaoJun Yang, Xin Guo, Linghui Du, Jian Yang

**Affiliations:** ^1^Department of Cardiology, The First College of Clinical Medical Sciences, China Three Gorges University, 443000 Yichang, Hubei, China; ^2^Institute of Cardiovascular Diseases, China Three Gorges University, 443000 Yichang, Hubei, China; ^3^Department of Cardiology, Qinghai Provincial People's Hospital, 810007 Xining, Qinghai, China; ^4^Department of Cardiology, The Central Hospital of Wuhan, Tongji Medical College, Huazhong University of Science and Technology, 430074 Wuhan, Hubei, China

**Keywords:** atherosclerosis, Nod-like receptor protein 3, T helper 17 cells, regulatory T cells, inflammatory factor

## Abstract

**Background:**

Regulatory T (Treg) cells are a class of anti-inflammatory lymphocyte subpopulations with a potential protective effect against atherosclerosis, whereas T helper 17 (Th17) cells have been reported to possess proatherogenic activity. It was believed that disturbed circulating Treg/Th17 balance was associated with the onset and progression of atherosclerosis. This study is designed to probe the regulative action of serum Nod-like receptor protein 3 (NLRP3) on the Treg/Th17 balance in patients with atherosclerosis.

**Methods:**

Fifty-two patients with coronary atherosclerosis and stenosis degrees of more than 50% were assigned to the coronary artery disease (CAD) group, and an equal number of people without coronary atherosclerosis were assigned to the control group (assessed by coronary angiography). Peripheral blood mononuclear cells (PBMCs) from two group patients were extracted and cultivated. The calculation of the Treg/Th17 ratio and quantitative analysis of the Treg and Th17 cell frequencies were performed through flow cytometry. Real-time fluorescence quantitative polymerase chain reaction (RT-PCR) was executed for the quantitative mRNA detection of the fork head-winged helix transcription factor (*Foxp3*) and the retinoic acid-related orphan nuclear receptor C (*RORC*) in PBMCs. Enzyme-linked immunosorbent assays were applied to measure the serum level of NLRP3, interleukin (IL)-10, IL-1β, IL-17A, IL-23, and transforming growth factor (TGF)-β1. Additionally, the connection between serum Treg/Th17 ratio and NLRP3 levels was analyzed using the Pearson correlation coefficient.

**Results:**

The baseline parameters, including sex, age, or blood biochemical indices had no difference in both groups (*p *> 0.05). The CAD group showed higher Th17 cell frequency, lower Treg cell frequency, and a lower Treg/Th17 ratio when compared to the control (*p *< 0.05). Consistent with the variation in the T-cell subset ratio, in patients with atherosclerosis, the Th17-cell-related transcription factor *RORC* showed a markedly higher mRNA level (*p *< 0.05), conversely, the mRNA expression of the Treg cell-related transcription factor *Foxp3* was notably reduced (*p *< 0.05). Similarly, the serum levels of NLRP3, IL-17A, IL-1, and IL-23 were significantly enhanced in CAD group but IL-10 and TGF-β1 were reduced (*p *< 0.05). Additionally, a negative correlation was found between NLRP3 and the Treg/Th17 ratio (r = –0.69, *p *< 0.001).

**Conclusions:**

Due to the potential impact on the serum Treg/Th17 ratio, NLRP3 may act as an aggravator in the onset and progression of atherosclerotic disease.

## 1. Introduction

Atherosclerosis is characterized as a chronic inflammatory disease with an intricate pathological process in which both innate and adaptive immunoinflammatory mechanisms are involved [1]. The inflammasome is known as an important component of innate immunity and can be activated by a series of pathogen- or injury-associated molecular patterns [2]. The Nod-like receptor (NLR) family of proteins represents a fourteen-member subset that acts as an essential component of the inflammasome, and its fourteen members are known as NLRP1~14. As a pattern recognition receptor (PRR) belonging to the NLR family, Nod-like receptor protein 3 (NLRP3) is directly induced by multiple external stimuli which activates cysteinyl aspartate specific proteinase 1 (caspase-1) from pro-caspase-1. Once activated, the latter leads to increased synthesis and secretion of interleukin (IL)-1β and other inflammatory factors by binding to the IL-1β precursor, thereby triggering the inflammatory response [3]. Helper T (Th17) cells and regulatory T (Treg) cells are two categories of T-cell subsets derived from CD4+ T cells that have been reported to possess immunosuppressive activity and proinflammatory activity, respectively [4]. The instability of Treg/Th17 balance is closely correlated with various autoimmune diseases infectious diseases, and tumors.

Since atherosclerosis is increasingly recognized as a chronic inflammatory disease, the crucial role of the interaction between multiple leukocyte subsets and inflammatory cytokines in atherogenesis has also been established. Similarly, the correlation between atherosclerosis and Treg/Th17 imbalance was recently revealed: under inflammatory conditions, the mutual transformation between Treg cells and Th17 cells is activated, which may lead to an alteration of the Treg/Th17 ratio and subsequently the promotion of atherosclerosis progression [5]. In addition, in our previous study, patients with acute myocardial infarction and coronary artery stenosis showed higher blood level of NLRP3, but the exact mechanism through which NLRP3 impacts atherosclerosis remains unclear. Interestingly, high expression of NLRP3 has been shown to promote the progression of inflammatory diseases by mediating the Treg/Th17 imbalance. Thus, we hypothesized that NLRP3 disturbs the Treg/Th17 balance by positively regulating the conversion of Tregs to Th17 cells, thereby promoting the overexpression of inflammatory factors and driving atherosclerotic plaque formation. The primary objective of this work was to investigate the potential impact of NLRP3 on the peripheral Treg/Th17 balance through the investigation and analysis of the correlation between serum Treg/Th17 ratio and NLRP3 level, intending to identify novel targets and a theoretical basis for atherosclerosis prevention and management.

## 2. Materials and Methods

### 2.1 Participated Patients

The coronary artery disease (CAD) group is constituted with 52 patients who were diagnosed with coronary atherosclerosis admitting by the Department of Cardiology at Wuhan Central Hospital and the First College of Clinical Medical Sciences at China Three Gorges University between January 2018 and December 2020. Following were the inclusion criteria: (1) one or more coronary arteries with more than 50% atherosclerosis diagnosed by coronary angiography and (2) no anti-inflammatory drugs or immunosuppressants for half a year. Moreover, the patients’ exclusion were performed as following criteria: (1) patients with severe cardiac-cerebral vascular diseases, such as stroke and myocardial infarction; (2) patients with hepatorenal insufficiency or other metabolic diseases; (3) patients with cancer; (4) patients with all kinds of acute and chronic infectious diseases; and (5) patients with a history of surgery within half a year.

The control group included 52 individuals who did not have coronary artery stenosis as determined by coronary angiography. The baseline parameters, including sex, age, or blood biochemical indices had no difference in both groups (*p *> 0.05), indicating comparability between the two groups (see in Table 1). All enrolled patients, as well as their family members, were made aware of this research and endorsed a written consent form for it. This study was under review and approval from the Medical Ethics Committee of Wuhan Central Hospital and the First College of Clinical Medical Sciences, China Three Gorges University.

**Table 1.** **Baseline characteristics in two groups**.

Characteristics	Control group	CAD group	*t/χ^2^* value	*p* value
	(n = 52)	(n = 52)		
Male [n (%)]	28 (53.8)	25 (48.1)	0.346	0.56
Current Smoking [n (%)]	16 (30.8)	22 (42.3)	–1.069	0.29
Hypertension [n (%)]	14 (26.9)	17 (32.7)	0.414	0.52
Age [($\bar{x}$ ± SD), (year)]	57.8 ± 6.9	59.3 ± 7.4	1.493	0.22
Fasting BG [($\bar{x}$ ± SD), (mmol/L)]	4.6 ± 0.4	4.5 ± 0.5	1.126	0.26
Serum Cr [($\bar{x}$ ± SD), (μmol/L)]	77.2 ± 17.3	80.6 ± 16.2	–1.035	0.30
TC [($\bar{x}$ ± SD), (mmol/L)]	4.1 ± 1.0	4.0 ± 0.9	0.536	0.59
TG [($\bar{x}$ ± SD), (mmol/L)]	1.5 ± 0.8	1.4 ± 0.7	0.678	0.50
HDL-C [($\bar{x}$ ± SD), (mmol/L)]	1.2 ± 0.2	1.3 ± 0.4	–1.613	0.11
LDL-C [($\bar{x}$ ± SD), (mmol/L)]	2.2 ± 0.8	2.3 ± 0.9	–0.599	0.551

Note: BG, blood glucose; Cr, creatinine; LDL-C, low density lipoprotein cholesterol; TC, total cholesterol; TG, triacylglycerol; HDL-C, high density lipoprotein cholesterol.

### 2.2 Measurement

All participants had their fasting venous blood samples drawn the morning of the day following admission. Density gradient centrifugation was performed to separate peripheral blood mononuclear cells (PBMCs) from the obtained blood sample. Resuspend the cell pellet with a moderate amount of phosphate buffer saline (PBS) and adjust the concentration of PBMCs to 2 × ${\mathrm{10}}^{6}$/mL using the blood cell counting plate. For Treg cells, the PBMC cell suspension was divided evenly into five tubes; then take five test tubes in which respectively add 100 μL PBMC cell suspension, 100 μL PBMC cell suspension, and 5 μL anti-human CD4 FITC antibody (Ebioscience, No. 85-11-0047-42), 100 μL PBMC cell suspension and 5 μL anti-human CD25 PE antibody (Ebioscience, No. 85-12-0259-42), 100 μL PBMC cell suspension and 5 μL anti-human CD127-PE-CY7 antibody (Ebioscience, No. 85-25-1278-42), and 100 μL PBMC cell suspension and all three mentioned antibodies (5 μL each), then label them as the blank tube, CD4+ T-cell single staining tube, CD25+ T-cell single staining tube, CD127-T-cell single staining tube, and CD4+ CD25+ CD127 Treg cell triple staining tube. Blend the mixture with precooling PBS and centrifuged horizontally (1500 r/min, 5 min) after incubation for 30 min (dark, 4 °C). After that, remove the supernatant and resuspend the reserved precipitate with PBS after washing twice for 3 min. For Th17 cells, incubate 100 μL PBMCs into a 24-well plate with 5 μL phorbol ethyl ester (Sigma, p1585, 25 ng/mL), 5 μL ionomycin (Sigma, 407951, 1 μg/mL), 5 μL monemycin (Solarbio, m8670, 1.4 μg/mL), and 5 μL brefeldin A (Sigma, b5936, 3 μg/mL) and culture in a 5% CO2 incubator at room temperature for 6 h. Consequently, collect the PBMCs and divide into evenly into five tubes. Then, take five test tubes and label them as the blank tube, CD3+ T-cell single staining tube, CD8-T-cell single staining tube, IL-17+ T-cell single staining tube, and CD3+ CD8-IL-17+ Th17-cell triple staining tube, in which add PBMC cell suspension (100 μL), PBMCs suspension (100 μL) and anti-human CD3 FITC antibody (5 μL, Ebioscience, No. 11-0039-42), PBMC cell suspension (100 μL) and anti-human CD8 APC antibody (5 μL, Ebioscience, No. 17-0088-42), PBMC cell suspension (100 μL) and anti-human IL-17A-PE antibody (5 μL, Ebioscience, No. 12-7178-42), and PBMC cell suspension (100 μL) and all three mentioned antibodies (5 μL each) respectively. The incubation and cell collection processes were the as above. Flow cytometry (Beckman Kurt Technology, model: CytoFLEX) was performed to examine the Th17 cell frequencies. RT‒PCR was performed to examine the mRNA expression of *Foxp3* and *RORC* The TRIzol method was performed to extract the total RNA of PBMCs. The cDNA synthesis kit given by Sigma Company offered instructions for reverse transcription of the RNA into cDNA. The SYBR premix Kit (Takara company) was employed for Real-time quantitative PCR. The following reaction conditions were adopted: 95 °C for 2 min, 95 °C for 15 s → 60 °C for 30 s → 72 °C for 30 s (40 cycles in total). The $2^{-\Delta \,\Delta \,\text{Ct}}$ comparative quantification method was used to analyze and process the results of real-time PCR. All primers for PCR amplification were synthesized or provided by Shanghai Shenggong Biology. *GAPDH* was used as the housekeeping gene in this experiment (Table 2).

**Table 2.** **Primers information**.

Gene	Sequence
*Foxp3*	Forward primer: 5′- AACAGCACATTCCCAGAGTTCC -3′
	Reverse primer: 5′- CATTGAGTGTCCGCTGCTTC -3′
*RORC*	Forward primer: 5′- CCGAGGATGAGATTGCCCTCT -3′
	Reverse primer: 5′- GGTGGCAGCTTTGCCAGGAT -3′
*GAPDH*	Forward primer: 5′-CCACATCGCTCAGACACCAT-3′
	Reverse primer: 5′-CCAGGCGCCCAATACG-3′

For another experiment, isolate serum from coagulated blood samples collected in nonanticoagulant tubes by centrifugation (3000 r/min, 20 min) min after 30min of standing. Collect upper serum and store it at –80 °C. Fasting blood glucose, blood lipids, and serum creatinine were measured by the laboratory department of Wuhan Central Hospital. The serum concentrations of NLRP3, IL-10, TGF-1, IL-1, IL-17A, and IL-23 were tested via the enzyme-linked immunosorbent test (ELISA). NLRP3 (Wuhan Feien Biotechnology, No. eh4202), IL-10 (Xinbosheng Biotechnology, No. ehc009.96), TGF-β1 (Xixinbosheng Biotechnology, No. ehc107b.96), IL-1β (Wuhan Feien Biotechnology, No. eh0185), IL-23 (Xixinbosheng Biotechnology, No. ehc171.96), and IL-17A (Xixinbosheng Biotechnology, No. ehc170.96) were detected according to the instructions of the enzyme-linked immunosorbent assay kit.

### 2.3 Statistical Analysis

IBM SPSS Statistics 22 (IBM Corp., Armonk, NY, USA) was used for all data analyses. Measurement data are presented with the formation of ($\bar{x}$ ± SD) and then compared through the *t*-test (2-tailed). Counting card information is presented as [n (%)] and compared with the χ^2^ test. The Pearson correlation coefficient was used to calculate the correlations. All statistical tests were two sided, and *p*-value of <0.05 was regarded as statistically significant.

## 3. Results

### 3.1 Comparison of Treg and Th17 Cell Frequencies in PBMCs and the Treg/Th17 Ratio of the Two Groups

Compared to the control group, individuals with coronary atherosclerosis had significantly lower Treg cell frequencies and Treg/Th17 ratios in their PBMCs (*p *< 0.05). Additionally, the Th17 frequency in PBMCs was increased 2-fold in the CAD group (Table 3).

**Table 3.** **Treg and Th17 Cell Frequencies and Treg/Th17 ratio. Data are means (±SD)**.

Group	n	Treg (%)	Th17 (%)	Treg/Th17
Control	52	6.1 ± 0.8	1.2 ± 0.3	5.1 ± 0.6
CAD	52	3.8 ± 0.6	2.3 ± 0.5	1.4 ± 0.3
t value		16.586	–13.604	39.774
*p *value		<0.001	<0.001	<0.001

### 3.2 The mRNA Expression of RORC and Foxp3 in PBMCs of the Two Groups

**Fig. 1.**
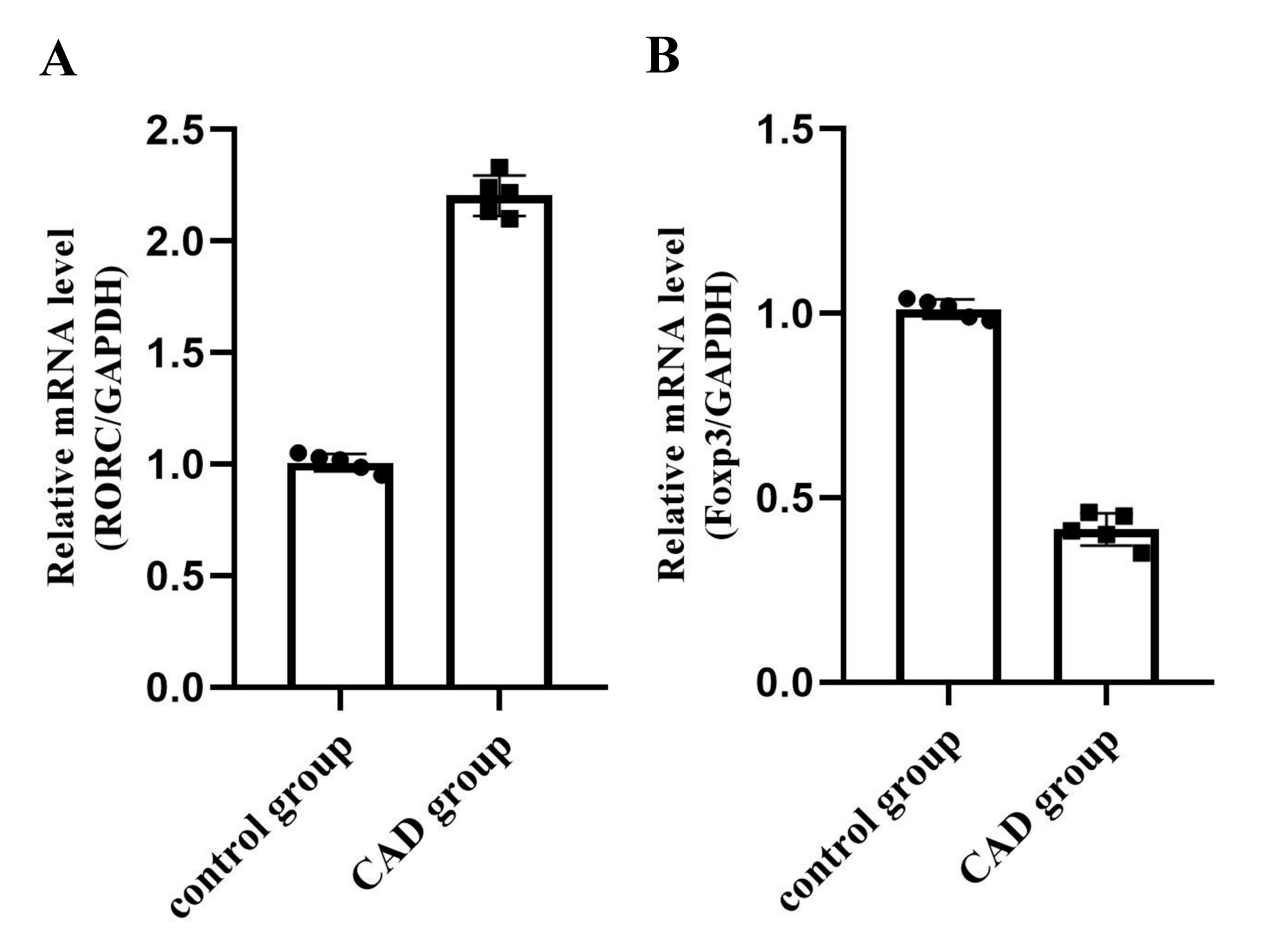
**The mRNA expression of *RORC* and *Foxp3* in PBMCs**. (A) In the CAD group, a considerably higher amount of *RORC* mRNA was found as compared to the control (n = 5, *p *< 0.05). (B) In the CAD group, a remarkably lower amount of *Foxp3* mRNA was showed as compare to the control (n = 5, *p *< 0.05).

*Foxp3* is known as a specific transcription factor which acts as a central effector in regulatory T cells growth and differentiation. *RORC* is regarded as a Th17 transcription factor. In support of those previously determined definitions of *Foxp3* and *RORC*, in this study, we observed a marked improvement of *RORC* mRNA expression and an obvious reduction of *Foxp3* mRNA in the CAD group (*p *< 0.05) (Fig. 1).

### 3.3 The Serum Levels of NLRP3 and Inflammatory Cytokines

As shown in Table 4, the serum concentrations of levels of NLRP3 and Th17 cell-related inflammatory cytokines (IL-1β, IL-17A, and IL-23) were remarkably higher in the CAD group when compared with control (*p *< 0.05). In contrast, the Treg cell-related anti-inflammatory cytokines (IL-10 and TGF-β1) in the CAD group were markedly lower than control (*p *< 0.05).

**Table 4.** **Serum level of NLRP3 and inflammatory cytokines. Data are means (±SD)**.

Group	n	NLRP3 (ng/mL)	IL-10 (pg/mL)	TGF-β1 (pg/mL)	IL-1β (pg/mL)	IL-17A (pg/mL)	IL-23 (pg/mL)
Control	52	2.2 ± 0.4	21.5 ± 4.3	472.5 ± 37.6	14.4 ± 3.8	19.1 ± 3.5	44.2 ± 5.2
CAD	52	5.6 ± 0.7	11.9 ± 2.9	235.5 ± 24.7	26.8 ± 5.5	54.6 ± 8.4	83.6 ± 7.2
t value		–30.411	13.347	37.989	–13.376	–28.131	–31.990
*p* value		<0.001	<0.001	<0.001	<0.001	<0.001	<0.001

Note: NLRP3, Nod-like receptor protein 3; IL-10, Interleukin 10; TGF-β1, Transforming growth factor beta 1; IL-1β, Interleukin 1 beta; IL-17A, Interleukin 17A; IL-23, Interleukin 23.

### 3.4 Correlation Analysis of the Treg/Th17 Ratio and NLRP3 Level in Serum of Atherosclerotic Patients

**Fig. 2.**
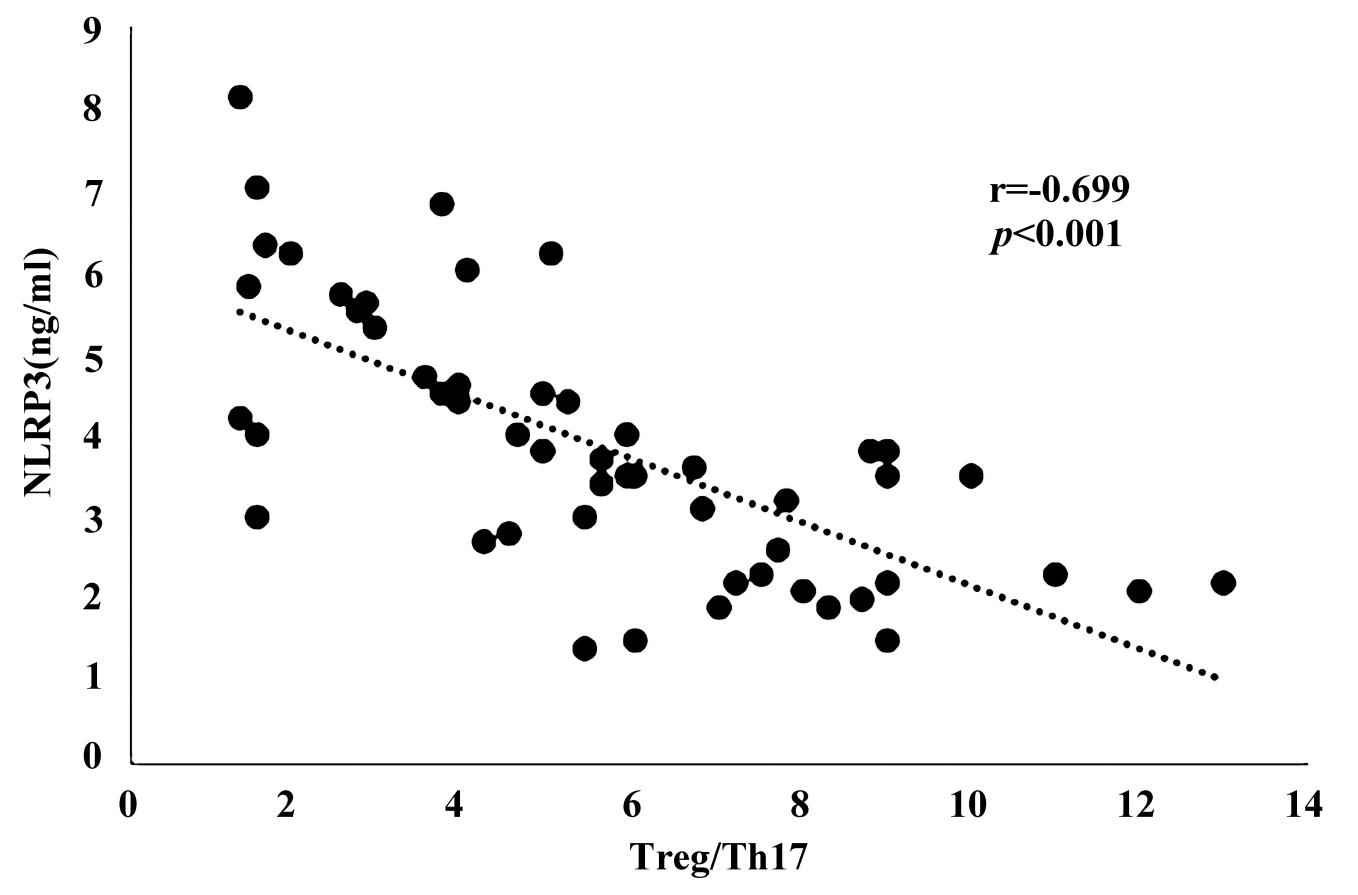
**Correlation analysis on serum NLRP3 level and Treg/Th17 in patients with coronary atherosclerosis (n = 52, r = –0.699, *p *< 0.001)**.

The negative correlation between the serum concentration of NLRP3 and the Treg/Th17 ratio is shown in Fig. 2 (n = 52, r = –0.699, *p *< 0.001).

## 4. Discussion

NLRP3 has been identified as a sensor component of the NLPR3 inflammasome that responds to various external stimuli and promotes inflammasome assembly [6]. The assembled NLRP3 inflammasome facilitates the cleavage and activation of caspase-1, which directly binds to the IL-1β precursor and triggers an immune response through the increased synthesis and secretion of inflammatory cytokines [3]. Additionally, the NLRP3’s activation also initiates the inflammatory cascade through the p65/NF-κB (NF-κB, nuclear factor kappa-B) pathway, then promotes the release of multiple proinflammatory cytokines including IL-6, TNF-α, IL-17, and IL-23. Upon secretion, proinflammatory cytokines in turn facilitate NLRP3 release by acting on activated monocyte-macrophages, thus forming positive feedback to prolong and maintain inflammatory responses [7, 8, 9]. However, although there is solid evidence of extracellular NLRP3 as a promoter of atherogenesis, the precise mechanisms of how NLPR3 impacts the formation and progression of atherosclerosis remain controversial [10, 11]. In the early stage of coronary artery disease, continued deposition of lipids and lipid-engorged cells in the arterial wall mainly contributes to the formation of atherosclerotic plaques [12]. Once activated, the increased expression of NLRP3 promotes the migration of monocytes and vascular smooth muscle cells into the injured arterial wall by positively regulating the synthesis and secretion of multiple inflammatory cytokines. This leads to the overloading of macrophages with lipids through the disturbance of lysosomes and the subsequent generation of foam cells and atherosclerosis progression [13, 14]. In this study, we reported an abnormally high level of serum NLRP3 in patients with coronary atherosclerosis, which roughly matches with those seen in earlier investigations, reconfirming the pathogenic potential of NLRP3 in atherogenesis [13, 14]. Besides, we also identified sevreal serum characteristics of immune disorder in atherosclerosis and furtherly linked NLRP3 with them: (i) In patients with atherosclerosis, the Th17 cell frequency and Treg-regulated anti-inflammatory cytokines was enhanced, whereas the Treg cell frequency, Th17-related inflammatory cytokines, and the Treg/Th17 ratio dropped. (ii) The serum NLRP3 level was inversely connected with anti-inflammatory cytokines but favorably associated with pro-inflammatory cytokines. (iii) The serum NLRP3 level was inversely associated with the Treg/Th17 ratio.

Interestingly, as two of the CD4+ T lymphocyte cell subsets, it has been thoroughly described in experimental animal research and clinical studies that Treg and Th17 cells exhibit crucial but diverse roles in atherosclerosis progression, in which Treg cells and related anti-inflammatory cytokines (TGF-β and IL-10) inhibit atherosclerosis progression, while Th17 cells and related pro-inflammatory cytokines exert the opposite effect [15, 16]. The Treg/Th17 imbalance has also been observed in patients with coronary heart disease and an animal model [5, 16]. Consistently, some alterations reflecting the Treg/Th17 imbalance and immune dysfunction were also reported in our study. While the serum Treg cell frequencies, mRNA expression of *Foxp3*, and serum levels of the anti-inflammatory cytokines TGF-β and IL-10 were significantly reduced in CAD group, markedly enhanced frequencies of Th17 cells were observed in patients with coronary atherosclerosis, accompanied by higher levels of *RORC* mRNA expression and the serum proinflammatory cytokines IL-1β, IL-17, and IL-23 (*p *< 0.05). These results concur with those of Potekhina, Wei, and other researchers’ work [17, 18]. Given that the immunologic feature of atherosclerosis always manifests as the upregulation of proinflammatory cytokines and downregulation of anti-inflammatory cytokines, it can be concluded from our results that Treg/Th17 imbalance and inflammatory disequilibrium engage in the onset and progression of atherosclerosis.

In addition, the regulatory effect of NLRP3 on the Treg/Th17 cell differentiation and its strong correlation with the progression of multiple immune diseases has been revealed in recent studies [19, 20, 21]. For instance, high expression of NLRP3 has also been observed in psoriatic lesions. Moreover, skin-specific NLRP3 suppression can inhibit the proliferation and chemotaxis of keratinocytes through the downregulation of IL-1β, IL-23, IL-17A, C-X-C motif chemokine ligand 1 (CXCL1), as well as the improvement of Treg/Th17 ratio, thus ameliorating the skin lesions induced by psoriasis [19]. However, this is the first study to link serum NLRP3 level with the Treg/Th17 ratio in atherosclerosis. We revealed a inverse association between serum Treg/Th17 ratio and NLRP3 level in individuals with coronary atherosclerosis, indicating that NLRP3 may act as a promoter in the initiation and progression of atherosclerotic plaques by modulating the Treg/Th17 ratio.

This study finds that serum NLRP3 level may reflect the Treg/Th17 ratio in individuals with atherosclerosis, however, some limitations remain existing. Significantly, the current findings of this study reveal a negative correlation between serum NLRP3 level and the ratio of Treg/Th17, which can be applied to the risk assessment of atherosclerosis. However, the negative correlation between NLRP3 and the Treg/Th17 ratio does not prove its causation, more evidence is required to examine NLRP3 as a key regulator for Treg/Th17 balance in further study. Moreover, the number of included patients with coronary atherosclerosis was only 52. Design of multicenter and a larger number of included patients might have provided more trustworthy results to support the correlation between serum NLRP3 and Treg/Th17 ratio.

## 5. Conclusions

In conclusion, the serum level of NLRP3 is inversely correlated with Treg/Th17 ratio in atherosclerosis. This finding provides a new idea for the research on immune mechanisms of atherosclerosis, clinical risk assessment of atherosclerosis and the prevention and treatment of atherosclerotic disease. Further investigations are required for the precise role and underlying mechanism of NLRP3 acting on the Treg/Th17 balance.

## Data Availability

The datasets used and/or analyzed during the current study are available from the corresponding author on reasonable request.
